# Structural Analysis and Characterization of an Antiproliferative Lectin from *Canavalia villosa* Seeds

**DOI:** 10.3390/ijms242115966

**Published:** 2023-11-04

**Authors:** Claudia F. Lossio, Vinicius J. S. Osterne, Vanir R. Pinto-Junior, Simin Chen, Messias V. Oliveira, Joost Verduijn, Isabel Verbeke, Sonia Serna, Niels C. Reichardt, Andre Skirtach, Benildo S. Cavada, Els J. M. Van Damme, Kyria S. Nascimento

**Affiliations:** 1 Laboratory of Biologically Active Molecules, Department of Biochemistry and Molecular Biology, Federal University of Ceara, Fortaleza 60440-970, Brazilbscavada@ufc.br (B.S.C.); 2 Laboratory of Biochemistry and Glycobiology, Department of Biotechnology, Ghent University, 9000 Ghent, Belgium; 3 Department of Physics, Federal University of Ceara, Fortaleza 60440-970, Brazil; 4 Nano-Biotechnology Group, Department of Biotechnology, Ghent University, 9000 Ghent, Belgium; 5 Glycotechnology Lab, Center for Cooperative Research in Biomaterials (CIC biomaGUNE), Basque Research and Technology Alliance (BRTA), Paseo de Miramon 194, 20014 Donostia-San Sebastián, Spain; 6 Centro de Investigación Biomédica en Red (CIBER-BBN), Paseo de Miramon 194, 20014 Donostia-San Sebastián, Spain

**Keywords:** *Canavalia villosa*, lectin, cancer, glycans

## Abstract

Cells use glycans to encode information that modulates processes ranging from cell–cell recognition to programmed cell death. This information is encoded within a glycocode, and its decoding is performed by carbohydrate-binding proteins. Among these, lectins stand out due to their specific and reversible interaction with carbohydrates. Changes in glycosylation patterns are observed in several pathologies, including cancer, where abnormal glycans are found on the surfaces of affected tissues. Given the importance of the bioprospection of promising biomolecules, the current work aimed to determine the structural properties and anticancer potential of the mannose-specific lectin from seeds of *Canavalia villosa* (Cvill). Experimental elucidation of the primary and 3D structures of the lectin, along with glycan array and molecular docking, facilitated the determination of its fine carbohydrate-binding specificity. These structural insights, coupled with the lectin’s specificity, have been combined to explain the antiproliferative effect of Cvill against cancer cell lines. This effect is dependent on the carbohydrate-binding activity of Cvill and its uptake in the cells, with concomitant activation of autophagic and apoptotic pathways.

## 1. Introduction

The biological information encoded in the compact structure of carbohydrates is efficiently used by cells to convey messages in the limited and crowded environment of the cell surface [1]. This glycocode results from the large structural variation that glycans are able to display. Cellular processes such as immune response, cell signaling, tissue development, hormone reception, and metastasis, among others, use this code to convey important biological information [2,3]. Effectively, the glycosylation pattern of membrane glycoconjugates transmits messages relating to the physiological state of a particular cell [4,5].

Aberrant glycosylation is frequently associated with cancer and metabolic diseases. Malignant tumors display notable alterations in tissue glycoconjugates and in the expression of enzymes involved in glycan biosynthesis or degradation [6]. Tumor cells tend to present a thicker glycocalyx due to increased production of glycans such as mucins and branched *N*-glycans. The synthesis of truncated glycan structures and extracellular acidification can often be observed in tumor microenvironments [7,8]. Some modifications in glycosylation patterns are detectable early in cancer development, and their progression is related to tumor growth [9]. Thus, altered glycans can potentially be used as targets for molecular biomarker identification, assisting in the process of early cancer diagnosis and treatment planning [6].

Since glycans are able to encode information in their structures, lectins are one of the main decoding molecules, capable of translating those messages into biological responses [3]. Lectins are defined as (glyco)proteins that bind mono- or oligosaccharides in a specific and reversible manner, without presenting catalytic activity in their carbohydrate-binding domain [10]. Lectins are present in every organism studied so far, playing different intrinsic roles. In plants, the interaction with carbohydrates is often directed towards exogenous sugars, suggesting functions as defense molecules or in plant symbiosis; moreover, new evidence supports its involvement in stress responses and signaling [11]. In animals, lectins act mainly by interacting with endogenous glycoconjugates, such as in cell adhesion and signaling processes [12]. Over the years, several lectins with different carbohydrate-binding specificities, different biological activities, and diverse structural profiles have been identified and isolated. Lectins are important biomolecules in glycobiology studies and display several biotechnological applications in medical research, including glycoproteomics research [13] and microarray construction [14], among other applications.

The interaction of lectins with typical cancer glycans has been studied since the early 1960s [15]. Some of these proteins have presented promising biological activities as they are able to bind differentially to tumors [16,17] or induce antiproliferative activities in cancer cells [18]. Generally, legume lectins induce cell death through mechanisms that involve autophagy and/or apoptosis with caspase activation. Apoptosis is an evolutionarily conserved cell death mechanism that is important in tissue development and which is triggered mainly by two mechanisms: the intrinsic, or mitochondrial, pathway and the extrinsic pathway, with the use of death receptors [19,20]. Autophagy is a finely regulated mechanism that works as an instrument for homeostasis-preservation and energy-maintenance purposes, recycling components that will be reused by the cells. On the other hand, the activation of autophagy can generate a high level of cellular stress, which in some cases ends up triggering cell death instead of cellular protection [21]. It is currently known that there is a crosstalk between autophagy and apoptosis, and both processes can play synergistic roles in the cell death process [22].

Cvill is a ConA-like mannose-specific lectin isolated from *Canavalia villosa* seeds (*Fabaceae* family, Phaseoleae tribe, Diocleinae subtribe). The biological properties and primary structure of the lectin have been partially determined in a previous work [23]. Small variations at the amino acid level are likely to exert significant influence on the efficacy and strength of legume lectins in various biological activities. A particularly noteworthy effect demonstrated by ConA-like lectins pertains to their potential as antineoplastic agents against a variety of cancer cell lines. This effect is consistently linked to their interaction with specific glycosylated receptors, which is a starting step for the activation of apoptotic and autophagic pathways [24] and this can happen concomitantly with lectin internalization in the cells through endocytosis, although this is not the case for many legume lectins [25]. To assess the potential of Cvill as an antiproliferative agent, viability assays were conducted on cervical carcinoma (HeLa), fibrosarcoma (HT1080), and fibroblast (NHDF) cell lines. In the case of the HeLa cell line, the assessment included the analysis of caspase activity, cell-death-specific mRNA expression, and ROS production to characterize the observed effects. Additionally, the primary and crystal structures of the lectin were determined, and the interaction with oligosaccharides and various glycans was investigated using glycan microarrays and molecular docking techniques, aiming to establish a correlation between the lectin’s structure and its biological activity.

## 2. Results

### 2.1. Cvill Primary Structure

**Figure 1:**
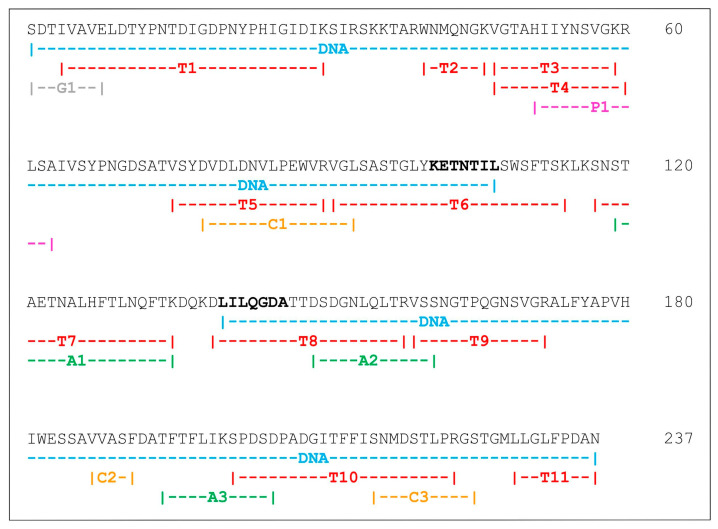
Amino acid sequence of the mature lectin from *C. villosa*. The primary structure was obtained through the combination of mass spectrometry and gene-sequencing data. The MS/MS peptide map and the region covered by DNA sequencing (in blue) are depicted under the amino acid sequence. Peptides were labeled according to the enzyme used for the proteolytic digestion, as follows: Trypsin (T, peptides in red), Chymotrypsin (C, peptides in yellow), Asp-N (A, peptides in green), Pepsin (P, peptides in purple), and Glu-C (G, peptides in gray). The regions used for primer design are shown in bold.

The lectin sequence was elucidated using a combination of mass spectrometry and gene sequencing. Lectin peptides were manually sequenced by interpreting mass spectra. MS/MS data alone covered 80% of the full sequence, with the remainder being covered by gene sequencing. An amplicon of approximately 660 bp was obtained and sequenced, generating two unique contigs (Supplementary Figure S1). Point differences in the nucleotide sequences between these two contigs confirmed that Cvill exists as multiple isoforms. Figure 1 displays the combination of both mass spectrometry and gene-sequencing data. Two conserved small fragments (Leu115–Lys116 and Asp137–Lys139) were not covered by either mass spectrometry or gene sequencing but were obtained by fitting to the electron density map (Supplementary Figure S2). Overall, after processing, the mature lectin structure is composed of 237 amino acid residues, similar to other ConA-like lectins [26].

BLASTp alignment indicated high sequence similarity with several *Canavalia* lectins. In particular, the metal-binding site of Cvill is highly conserved, consisting of residues Glu8, Asp10, Tyr12, Asn14, Asp19, and His24 (Supplementary Figure S3). An unexpected variation has been noted in the CRD, where Methionine at position 228 replaces the typical Arg228.

### 2.2. Crystal Structure

The structure of the lectin complexed with α-methyl-D-mannopyranoside (α-mm) was determined through macromolecular crystallography and X-ray diffraction. Crystal growth was achieved using Condition A6 from the JBScreen JCSG++ kit, which consists of 20% PEG1000, 100 mM potassium phosphate citrate at pH 4.2, and 200 mM lithium sulfate. Crystals were successfully nucleated after a relatively short incubation period of 2 weeks at room temperature. Further optimization of this condition (17.5% PEG1000, 0.1 M potassium phosphate citrate pH 4.2 and 200 mM lithium sulfate) resulted in the formation of suitable crystals for diffraction. The crystals adopted the I222 orthorhombic space group with unit cell parameters of a = 61.49 Å, b = 84.44 Å, and c = 89.07 Å, and one monomer was found in each asymmetric unit. Additional information regarding data collection, molecular replacement, and refinement parameters is available in Table 1. The X-ray structure was solved through molecular replacement using the coordinates of the *Canavalia grandiflora* lectin (ConGF) (PDB: 4L8Q). Ligand fitting and refinement were carried out using Coot and Phenix. The model’s validity was confirmed using the PDB validation tool and has been deposited in the PDB database under the identification code 8SZO.

**Figure 2:**
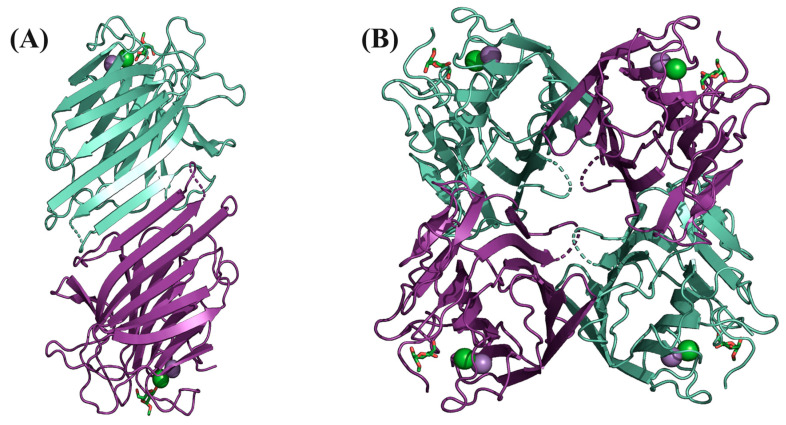
Three-dimensional structure of *C. villosa* lectin. (**A**) Dimer representation. The 12 aligned β-strands are shown in the front. (**B**) Cvill homotetramer. The structures are in cartoon representation with monomers in green and purple.

Structurally, Cvill exhibits the typical legume lectin fold, also known as a β-sandwich, characterized by the presence of two superimposed beta sheets, resembling a sandwich. These beta sheets consist of antiparallel strands, with one beta sheet formed by six long, flat beta strands and the other by seven curved strands [27]. The arrangement of dimers and tetramers in the quaternary structure was predicted using the web-based interactive tool PISA (EMBL-EBI) and involves non-covalent interactions between the β-sheets of two different monomers. The canonical quaternary structure of Cvill is a tetramer, formed by the 222 point group symmetry in the space group I222 of the crystal, as depicted in Figure 2.

Residues 117–123, located in the loop region, have also been suggested to play a role in stabilizing the tetramer [28]. In the current lectin, six residues from this loop (117SNS-TAE122) were not modeled due to poor electron density in this region. Poor electron density data in this region are often observed in other ConA-like lectin structures solved through macromolecular crystallography and are attributed to the high flexibility that leads to different arrangements during X-ray diffraction.

#### Cvill-Binding Sites

**Figure 3:**
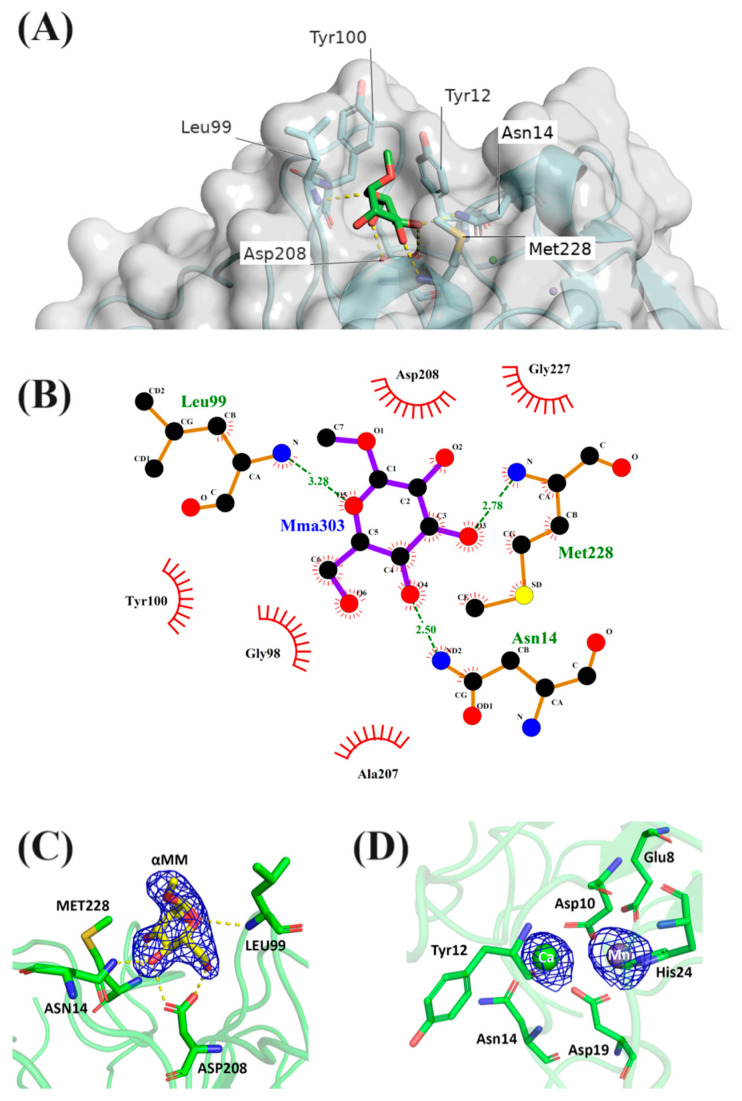
Carbohydrate-Recognition Domain (CRD) of Cvill. (**A**) Three-dimensional representation of the lectin-binding site occupied by the ligand α-methyl-mannopyranoside (in green). (**B**) LIGPLOT representation of the hydrogen bonds and the hydrophobic interactions established between α-methyl-mannopyranoside (Mma303) and the CRD. (**C**) Electron density map and resulting atomic model of the ligand (in yellow) in the CRD. (**D**) Ca^2+^ and Mn^2+^ within the metal-binding site.

The CRD of Cvill is composed of four loops: Thr11-Pro23, Ser96-Asn104, Leu198–Asp208, and Ser225–Leu232. Residues Asn14, Gly98, Leu99, Asp208, and Met228 directly interact with atoms O3, O4, O5, and O6 of the ligand through polar interactions (see Table 2). The backbone nitrogen of residues Leu99 and Met228 also forms direct hydrogen bonds with the oxygens O5 and O3 (see Figure 3A,B). The carbohydrate-binding pocket, calculated using the PyVOL plugin, has a relatively large volume of 205.0 Å^3^.

Residues participating in the metal-binding site are conserved, with the calcium ion interacting with Asp10, Tyr12, Asn14, Asp19, and two water molecules. Similarly, the manganese ion interacts with residues Glu8, Asp10, Asp19, and His24, along with two water molecules (see Supplementary Figure S4). These residues in the metal-binding site are symmetrically arranged in an octahedral geometry and are spatially located in the vicinity of the CRD. The coordination of Asp208 through a water bridge by the calcium ion stabilizes the “cis-peptide” conformation typically found between residues Ala207 and Asp208 of legume lectins, thereby assisting in the correct arrangement of the CRD conformation [29].

### 2.3. Carbohydrate-Binding Analysis

**Figure 4:**
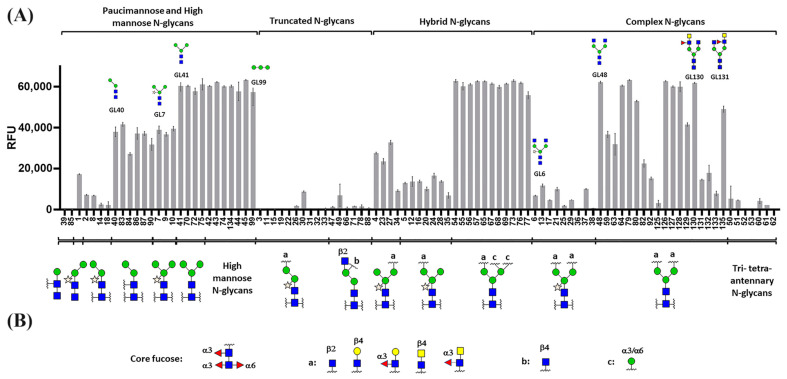
Glycan array incubation of Cvill. (**A**) Histograms representing mean RFU (Relative Fluorescence Units) values after incubation with fluorescently labeled Cvill. Each histogram represented the averaged RFU values for four replicates with the standard deviation of the mean. (**B**) Legend showing the different substituents of the *N*-glycan backbones. Structures printed on the microarray are presented in Supplementary Figure S5. The structure of the glycans is represented in the standard Symbol Nomenclature for Glycans (SNFG) v. 1.5.

Glycan array experiments indicated that Cvill exhibits affinity for terminal α-mannosyl residues and the tri-mannosidic core of *N*-glycans. Figure 4 presents the glycan-array-binding data for Cvill, grouped by the type of *N*-glycan, including pauci- and high-mannose glycans, truncated, hybrid, and complex structures. Based on the binding profile, we identified the paucimannosidic tetrasaccharide 40 (Manα-1,3-Manβ-1,4-GlcNAcβ-1,4-GlcNAc) as the smallest binding structure on our array. The presence of an additional Manα-1,6-Man branch, which generates the pentasaccharide core of *N*-glycans (glycan 41), increased the interaction, as observed for all high-mannose *N*-glycans on the array. The trimannoside Manα-1,2-Manα-1,2-Man (glycan 99), found in the Man9 structure, also exhibited strong interaction with the lectin.

Conversely, truncated *N*-glycans lacking the Manα-1,6-Man but displaying different substitutions such as GlcNAc, Galβ-1,4-GlcNAc, and GalNAcβ-1,4-GlcNAc on the 3-arm were not recognized. Similarly, hybrid *N*-glycans with terminal α-mannosyl residues displayed particularly strong binding. Biantennary complex *N*-glycans bound Cvill in most cases, irrespective of the presence of terminal GlcNAc, Gal, GalNAc, or core fucosylation. However, the presence of β-1,2-xylose reduced lectin binding, as observed when comparing structures 7 vs. 41 and 6 vs. 48. Multi-antennary complex *N*-glycans with three or more branches weakly interacted with the lectin, likely due to larger steric hindrance, resulting in more limited access to the mannose core.

On the immobilized glycans in our array, Cvill exhibited a binding preference for positional isomers with a GalNAc residue on the 3-branch (glycan 130) over the 6-branch (glycan 131).

**Figure 5:**
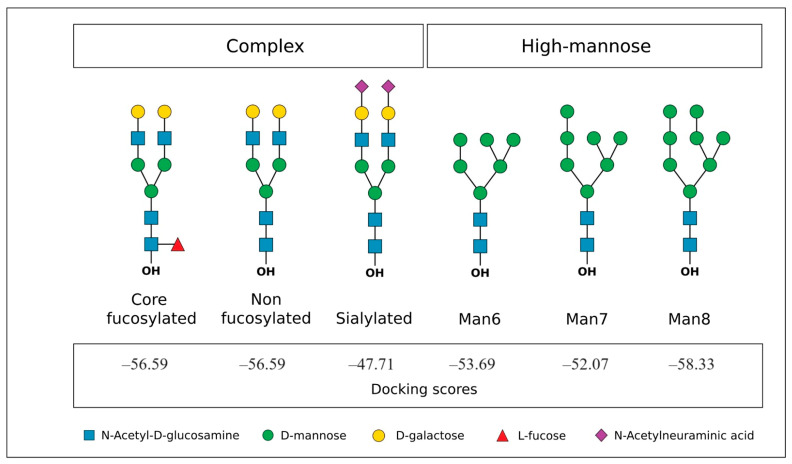
HeLa *N*-glycans tested in molecular docking simulations and docking scores. Glycans are represented in the standard Symbol Nomenclature for Glycans (SNFG) v. 1.5.

Molecular docking experiments focused on the interaction of Cvill with glycans known to be present in the glycocalyx of cancer cells, such as HeLa cells. Therefore, complex *N*-glycans, whether core-fucosylated or not, and the high-mannose *N*-glycans Man6, Man7, and Man8 were selected for in silico binding prediction experiments [30]. All tested glycans were efficiently recognized when compared to α-mm. Scores ranged from −43.71 to −58.33 (see Figure 5). Fucosylated and non-fucosylated complex *N*-glycans bound well and presented similar scores, confirming the data from the glycan array.

**Figure 6:**
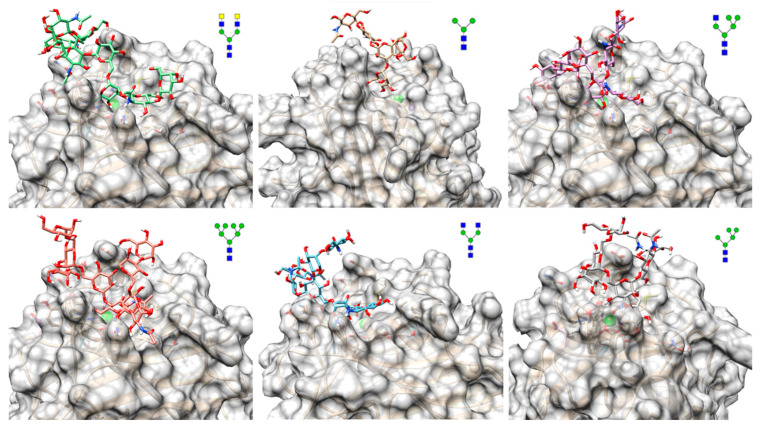
Docking poses of the interaction between Cvill and representative glycans from the array. The lectin is represented as cartoons (in gold) with overlaid surfaces (in gray) and the ligands are represented as sticks with carbons in varying colors. The structure of each glycan can be seen in Symbol Nomenclature for Glycans (SNFG) v. 1.5.

To further evaluate the binding mode of glycans within the lectin-binding site, molecular docking experiments were conducted using representative structures from the glycan array to which the lectin exhibited a favorable binding affinity (refer to Figure 6). Simulations revealed a strong preference for the Manα-1,3-Man branch of the mannose core, a substructure consistently found within the CRD for all tested glycans. Additionally, the simulations suggested that Cvill can provide an extended binding site capable of accommodating additional residues beyond those responsible for monosaccharide interactions, leading to stable interactions when the ligand is larger.

### 2.4. Cell Viability Assays

**Figure 7:**
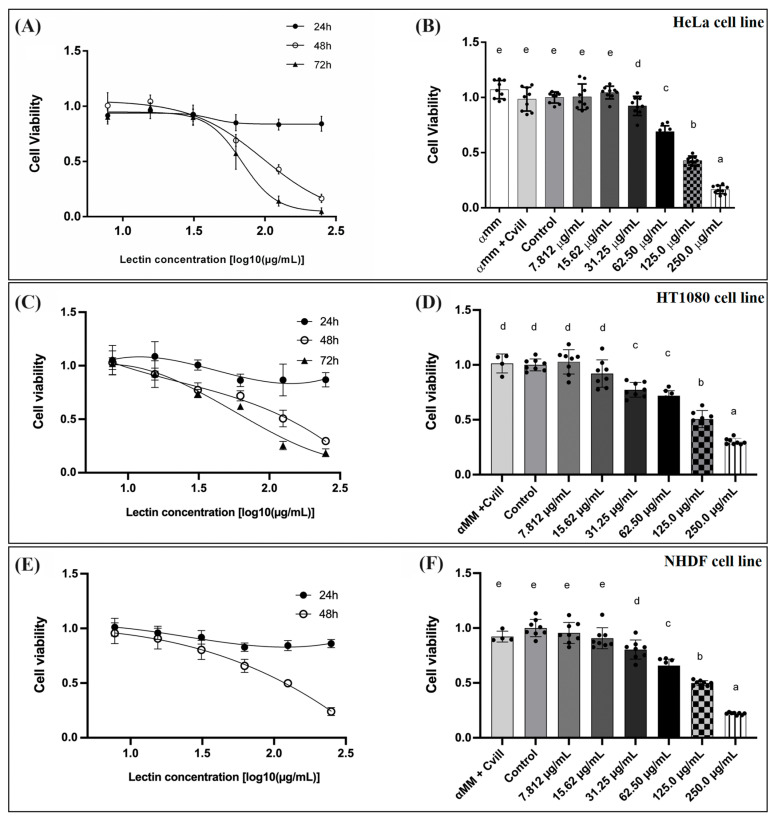
Evaluation of Cvill cytotoxicity on various cell lines. (**A**) Dose–response curves illustrating the cytotoxicity of Cvill against HeLa cells after 24, 48, and 72 h of incubation. (**B**) Bar chart presenting the results of the 48 h experiment on HeLa cells. (**C**) Dose–response curves depicting the cytotoxicity of Cvill against HT1080 cells after 24, 48, and 72 h of incubation. (**D**) Bar chart representing the outcomes of the 48 h experiment on HT1080 cells. (**E**) Dose–response curves demonstrating the cytotoxicity of Cvill against NHDF cells after 24 and 48 h of incubation. (**F**) Bar chart illustrating the results of the 48 h experiment on NHDF cells. Cell viability is expressed as the ratio of cytotoxic responses to the control and the inhibition of lectin toxicity by its specific sugar, α-methyl-mannopyranoside, is included in the bar charts. Data are presented as the average ± SD, based on three experiments in triplicate for HeLa cells and two experiments in quadruplicate for HT1080 and NHDF cells. Significance was assessed using one-way ANOVA and Tukey’s multiple comparisons test with a 95% confidence interval. Conditions within the same cell line that do not share lowercase letters are significantly different.

Considering the specificity of Cvill for *N*-glycans present on HeLa cells, the cytotoxicity of this lectin was evaluated. The lectin demonstrated dose-dependent cytotoxicity against all tested cell lines (Figure 7A,C,E) and this toxicity was carbohydrate-mediated (Figure 7B,D,F). Cvill presented IC_50_ values of 97.0 μg/mL after 48 h incubation period (R^2^ = 0.95). Cvill also affected the viability of the fibrosarcoma (HT1080 cell line) and the NHDF cell line with IC_50_ values of 116.08 μg/mL (R^2^ = 0.96) and 108.34 μg/mL (R^2^ = 0.94) after a 48 h incubation period. In addition to a reduction in viability, the lectin altered the cell morphology of all tested cell lines, changing their round shape and, at higher concentrations, Cvill induced the loss of adhesion and agglutination (see Supplementary Figure S6). The addition of 100 mM α-methyl-D-mannopyranoside to the medium prior to the assays completely blocked the cytotoxic effect of Cvill for all cell lines studied.

**Figure 8:**
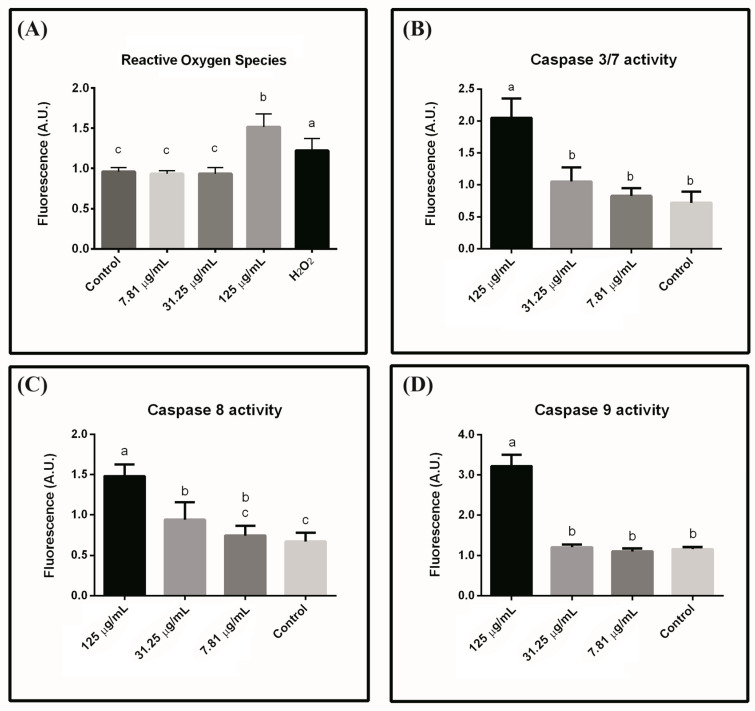
ROS production and caspase activity. (**A**) Analysis of the Reactive Oxygen Species (ROS) produced after 48 h incubation of HeLa cells with Cvill at concentrations 125.0 μg/mL, 31.25 μg/mL, and 7.81 μg/mL. Hydrogen peroxide was used as a positive control. (**B**–**D**) The activity levels for caspase 3/7, 8, and 9, respectively, after 48 h incubation of HeLa cells with Cvill. Each bar represents the mean ± SD of three different experiments in triplicate. Bars labeled with different lowercase letters indicate statistically different data (*p*-values < 0.01). Significance was assessed using ANOVA and Tukey’s multiple comparisons test.

To understand the pathway by which the lectin induces cytotoxicity, ROS levels and caspase activation were analyzed for HeLa cells. The highest lectin dose (125 μg/mL) led to intracellular ROS accumulation, while cells incubated with the two lowest doses did not show ROS production (Figure 8A). This result suggests that incubation with the lectin at 125 μg/mL induced a state of oxidative stress. Caspase assays revealed that the activity of all four cysteine proteases increased at the highest lectin dose (125.0 μg/mL) (see Figure 8B–D). At an intermediate dose (31.25 μg/mL), only caspase 8 increased, suggesting that, at this dose, apoptosis was initiated only through the non-mitochondrial (extrinsic) pathway. In contrast, no caspase activity was observed at the lowest lectin dose.

qRT-PCR experiments further allowed the evaluation of transcript levels of genes involved in apoptosis (Bax, Bak, FADD, and PUMA) and autophagy (LC3) in HeLa cells treated with Cvill (125.0 μg/mL, 31.25 μg/mL, and 7.81 μg/mL) for an incubation period of 48 h. Transcript levels for Bax, Bak, and FADD were downregulated at the highest lectin dose, while PUMA was significantly upregulated in HeLa cells treated with high and intermediate lectin doses. Additionally, LC3 transcripts increased at high lectin concentrations (see Supplementary Figure S7).

### 2.5. Internalization of Cvill in HeLa Cells

**Figure 9:**
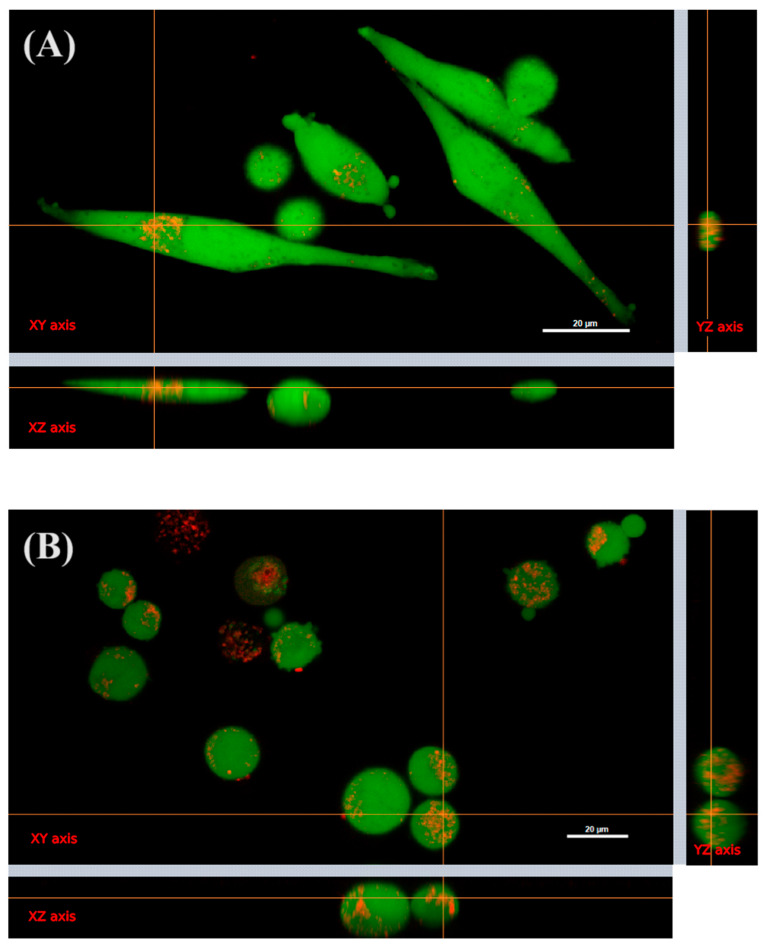
Confocal microscopy of HeLa cells incubated with 31.25 μg/mL (**A**) and 125.0 μg/mL (**B**) Cvill. To represent the three-dimensional perspective of confocal images, the orthogonal views of planes XY, XZ, and YZ were arranged for each image. The internalization of the lectin by the cell is observed in both lectin concentrations tested. Cvill was labeled with TRITC (red band emission—560 nm), HeLa cells were stained with calcein (green—488 nm), and 60× magnification objective lenses were used.

Confocal microscopy images showed that the lectin is internalized into HeLa cells after an incubation period of 48 h (Figure 9). The lectin accumulates near the nucleus but it is not clear whether Cvill is accumulating inside a specific organelle.

## 3. Discussion

The lectin isolated from *Canavalia villosa* seeds, Cvill, is a mannose-specific non-glycosylated ConA-like lectin, which undergoes circular permutation and is composed of a single polypeptide chain with 237 amino acid residues. The Cvill monomer exhibits the canonical legume lectin fold and assembles into a tetrameric structure resembling the ConA dimer-of-dimers [31].

Although the Cvill sequence is highly similar to other ConA-like lectins, a striking difference was observed at position 228, an important residue for carbohydrate interaction, where a methionine was present instead of the usual arginine. Analysis of the structure indicates that Met228 interacts with the ligand through its backbone nitrogen, similar to Arg228 in similar lectins. The number of interactions computed via the CONTACT software v. 1.12.88 suggests that the binding of Cvill to α-mm is stable (Table 2). Usually, lectins that have Arg228 in proximity to residues Asn14 and Tyr100 present a wide and shallow CRD. Different CRD geometries have been correlated with distinct vasorelaxant properties of ConA-like lectins, with wider CRD geometries correlating with stronger binding to ligands [26]. Other ConA-like lectins complexed with α-mm presented smaller CRD volumes compared to Cvill (205.0 Å^3^), such as ConA (PDB: 5CNA) with a volume of 175.0 Å^3^ and *Canavalia bonariensis* lectin (CaBo) (PDB: 5U3E) with a volume of 161.0 Å^3^. The larger binding site of Cvill is possibly a consequence of the Met228 substitution and its proximal positioning.

Considering that the anticancer effect of Cvill is dependent on the interaction with glycosylated receptors at the cell surface, the binding of the lectin with structurally complex carbohydrates was analyzed through glycan array and molecular docking. The glycans tested for docking included those found on HeLa glycocalyx and structures from the glycan array library [30,32,33]. Metastatic cells and cervical cancer cells present a glycocalyx rich in high-mannose *N*-glycans. The aberrant glycosylation pattern found in tumor cells is considered a hallmark of cancer and results from altered expression of glycosyltransferases and glycosidases. Since it was expected that Cvill has typical ConA-like specificity towards high-mannose glycans, docking experiments focused on the interaction with these typical mannose-rich tumor markers. Glycan microarray experiments revealed that the lectin binds strongly to mannose-rich *N*-glycans; Cvill also exhibited the expected specificity towards the core trimannoside of other *N*-glycan types. This trimannoside core is efficiently recognized even when substituted with different terminal epitopes.

Taking into account the array results, the binding of Cvill to glycan structures was further tested through molecular docking. The simulations suggested that, for most glycans, the Manα-1,3-Man branch of the trimannose core often occupies the CRD, in agreement with the glycan array results. The combined binding data indicated the importance of the mannose core for the lectin binding. Higher levels of branching in complex glycans and xylosylation on the first mannose after the initial *N*-acetyl-chitobiose weaken the interaction due to steric hindrances. Previous data for ConA-like lectins corroborate the current results [34,35]. Additionally, array experiments demonstrated that Cvill was able to selectively bind biantennary glycans with asymmetrical branches, as the binding intensity was noticeably different depending on whether fucose and/or GalNAc residues were added to branch 3 or 6 of the *N*-glycan.

The extensive binding of Cvill to mannosylated glycan structures, glycans found on the HeLa cell surface and a similar binding profile to other ConA-like lectins justified its application in viability assays against different cancer cell lines. Several mannose-specific lectins have been reported to efficiently bind the altered glycocalyx of cancer cells and induce cytotoxic responses [24,36]. One of the major modifications found in metastatic cells involves hyperglycosylation and the early termination of *N*-glycan processing, which can generate high concentrations of unusual glycan structures [37]. The cytotoxic activity of legume lectins on neoplastic cells has been documented and varies according to the lectin and/or the cell line used. ConA has proven to be toxic in different cell cultures, with IC50 values of 15 μg/mL in human breast carcinoma (MCF-7) [38], 5 μg/mL in hepatoma (HepG2) [39], 3 μg/mL in leukemia (MOLT-4) [16], and approximately 40 μg/mL in cervical carcinoma (HeLa) [40], while *Canavalia virosa* lectin (ConV), which shares 91.14% identity with Cvill, had an IC50 of 100 μg/mL in glioma cells (C6) [41].

Cvill induced cytotoxicity in HeLa, HT1080, and NHDF cell lines, and, as hypothesized, the effect is dependent on the lectin binding to mannosylated receptors on the cell surface, since the presence of α-methyl-D-mannopyranoside in the medium completely abolished the cytotoxic effect. Cvill presented a stronger effect against HeLa cells, in comparison to HT1080 and NHDF cell lines, this being compatible with the higher high-mannose content on the cell surface of HeLa cells compared to the other cell lines [30,42,43]. To gain deeper insights into the cytotoxic effects of Cvill, a mechanistic analysis was conducted for lectin-treated HeLa cells. Overall, the lectin appears to induce cytotoxicity through intrinsic and extrinsic apoptosis and autophagy mechanisms. This is suggested due to high levels of caspase activity, upregulation of PUMA, and LC3. Since caspases 8 and 9 are related to extrinsic and intrinsic apoptosis pathways, respectively, it can be inferred that both apoptotic pathways might have been activated [44,45]. For intrinsic apoptosis, 125.0 μg/mL Cvill triggered the activation of caspase 9, which is related to the activation of effector caspases 3 and 7, release of cytochrome C from the mitochondria, and increased production of ROS, indicating oxidative stress [46]. Upregulation was observed for both PUMA and LC3, which are associated with the activation of the autophagic pathway. PUMA’s role is to prevent the anti-apoptosis proteins Bcl-XL and Bcl-2 from binding to Beclin1, an inducer of autophagy that can be inhibited by Bcl-2 superfamily proteins [47]. On the other hand, LC3 is a protein linked to the microtubule network, involved in the maturation of the autophagosome and the subsequent initiation. High expression of PUMA, as seen in qRT-PCR experiments, is also associated with intrinsic apoptosis, as this protein is a member of the Bcl-2 superfamily and binds to the anti-apoptotic proteins Bcl-XL and Bcl-2, leaving Bak and Bax free to act in the formation of pores in the mitochondria, thus triggering intrinsic apoptosis [48]. Cvill treatment also resulted in Caspase 8 activation at the tested doses. For the higher lectin dose, the extrinsic pathway occurred simultaneously with the activation of intrinsic apoptosis and the autophagic route, while the intermediary dose of Cvill only induced caspase 8 activation, with no production of ROS or activation of other caspases.

In summary, Cvill interaction with glycoconjugates in the cell surface is the starting step for the activation of both intrinsic and extrinsic apoptosis, as well as autophagic mechanisms at the higher lectin dose. However, at the intermediary lectin dose, only extrinsic apoptosis was observed. This aligns with what has been observed for ConA and ConBr, where necrosis was triggered at high lectin concentrations in leukemia cells, while apoptosis occurred at lower concentrations [16]. In the case of the HeLa cell line, ConA induced cell death through apoptosis and autophagy with longer incubation periods, while it primarily induced autophagy with shorter exposure to the lectin [40,49]. Furthermore, ConA induced autophagy in hepatoma cell lines, glioma cells, and HeLa cells [40,49,50].

The downregulations of FADD, Bax, and Bak expression with the highest lectin dose gave further insight into Cvill antiproliferative mechanism. The Fas-associated death domain protein (FADD) acts as a universal death receptor adapter. Usually, caspase 8 activation is associated with an increase in FADD expression. Once death-inducing signals, such as the cytokine TNF-α, bind to the Death Receptor (DR), FADD transduces the death signal intracellularly through the formation of the Death-Inducing Signaling Complex (DISC), activating caspase 8 and initiating extrinsic apoptosis [45,51]. The downregulation of FADD might indicate that caspase 8 activation was not dependent on DR binding. It has been reported that the interaction of galectin-3 with the *N*-glycan of the Fas death receptor triggered apoptosis by the extrinsic pathway, activating Caspase 8, while ConA was reported to be able to effectively bind the glycans from the Fas receptor [52,53]. However, the downregulation of FADD after treatment with Cvill might indicate that the lectin did not interact with a receptor from the TNFR family to trigger Caspase 8 activation but rather interacted through a mechanism similar to the lectin from *Viscum album*, which induced Caspase 8 activation in human leukemic cell lines due to lectin internalization, independently of death receptors [54]. This hypothesis is strengthened by confocal analysis with TRITC-labelled lectin, which provided evidence for internalization of the lectin in HeLa cells.

The cytotoxicity against all tested cell lines occurred concomitantly with morphological changes resembling the anoikis process. Anoikis is a variety of apoptosis in which programmed cell death is triggered by the loss of cell interaction with the extracellular matrix. The induction of this process is an excellent approach in cancer therapeutics since it can help to prevent detached cells from surviving in the circulatory system and invading other healthy tissues, which may result in metastasis [55]. Cvill-treated cells lost their adhesion and became spherical in shape. Anoikis has been reported to be triggered by the galactose-specific lectin from *Morus alba* in breast cancer cells [56], and HeLa cells treated with frutalin, a galactose-specific lectin from *Artocarpus incisa*, presented anoikis-like morphology as well [57]. There is evidence that shows that strong caspase 8 activation is related to cell detachment and the anoikis process [58]. Apoptosis and autophagy mechanisms can be initiated both by binding to extracellular receptors and after lectin incorporation by the cell. Similar to Cvill, the mannose-specific lectin from *Sophora flavescens* was incorporated by HeLa cells during the process of cell death. In hepatoma cells [59], ConA was internalized and accumulated in mitochondria, where it triggered autophagy in hepatoma cells [39]. Likewise, the previously mentioned lectin from *V. album*, which induced extrinsic apoptosis independent of cell receptors, showed toxicity after being internalized [54]. Other mannose-specific lectins such as artocarpin (*Artocarpus integrifolia* lectin) and MornigaM (*Moringa oleifera* lectin) were also rapidly endocytosed by lymphocyte cells during cytotoxicity assays [60].

The binding of exogenously applied lectins to glycans on cancer cells is typically considered an important step, but by itself it is insufficient for the activation of cell death processes [24]. Lectin binding to specific receptors must occur, and can result in lectin internalization, further activating cascades that lead to apoptosis through the mitochondrial pathway [50,61]. One of the best-researched molecular targets for plant lectins is Membrane-Type Matrix Metalloproteinase-1 (MT1-MMP), a glycosylated protein crucial for metastasis and neovascularization in tumor cells [50,62]. Another prominent receptor for mannose-binding lectins is the Protein Zero-Related protein (PZR), a highly glycosylated transmembrane receptor that may be involved in detachment and migration of adherent cells. The glycosylation profile of this receptor is known to vary depending on the cancer type [63]. In addition to these, several Receptor Tyrosine Kinases (RTKs) and Toll-like receptor 2/6 (TLR-2/6) have been shown to be capable of binding mannose-binding legume lectins [24]. To date, there is no consensus regarding the exact target for the antiproliferative activity of legume lectins. However, it is more likely that the anti-cancer effect of these lectins is elicited through binding with multiple receptors simultaneously.

In vitro experiments are an initial step towards in vivo experiments; however, important differences exist between cell cultures and animal models. ConA-like lectins are known to be potent mitogenic effectors for specific normal cell types depending on the dose [24]. These lectins are also immunomodulatory agents inducing cytokine release; however, the latter is a double-edged sword since liver injury is a serious side-effect of the cytokine storm elicited by these lectins when applied in vivo. The cytokines include pro-inflammatory (TNF-α, IFN-γ, IL-1, IL-6, and IL-12) and anti-inflammatory (IL-4, IL-10, and IL-22) cytokines [64]. However, at lower dosages, the liver damage can be fully avoided, and the cytokine response can be shifted towards a beneficial anti-inflammatory response [65].

Besides the standalone use of the lectin in a systemic or local route, perhaps the most promising approach for utilizing Cvill involves using nanoparticles to achieve controlled lectin release within the tumor area or incorporating the lectin into drug-delivery systems [18,66]. This leverages its preferential binding to malignant cells to guide nanostructured systems containing conventional drugs to the tumor, thereby reducing undesired side-effects [67,68]. Additionally, Cvill can serve as a tool for glycomics and glycoproteomics studies, facilitating the investigation and detection of glycosylation profiles on different cancer cell lines by exploiting the various glycans present on the cell surface [69,70,71]. This enables the use of the lectin as a diagnostic tool and in cancer biomarker research.

## 4. Materials and Methods

### 4.1. Lectin Purification

Cvill was purified from seeds of *Canavalia villosa* following the protocol described by Lossio et al. (2017) [23]. Lectin purity was studied via SDS-PAGE [72] and its activity was confirmed via agglutination assays using a 2% suspension of rabbit red blood cells.

### 4.2. Sequence Determination

The complete amino acid sequence of Cvill was determined using a combination of mass spectrometry and gene sequencing as summarized below.

#### 4.2.1. Electrospray Ionization Mass Spectrometry (ESI-MS/MS)

The polypeptide corresponding to the α subunit of Cvill was excised from SDS-PAGE gels and digested according to Shevchenko’s protocol [73]. The protein was digested with trypsin, chymotrypsin, pepsin, Asp-N, and Glu-C, and the resulting peptides were extracted in a 5% formic acid in acetonitrile (1:1) solution. Analysis was performed in a Q-TOF Premier Mass Spectrometer (Waters Corporation, Milford, MA, USA) combined with a NanoAcquity UPLC liquid chromatography system. Peptide injection was performed through a C-18 reverse phase matrix (100 μm × 100 mm) with a flow rate of 600 nL/min and elution taking place with an increasing gradient of acetonitrile in 0.1% formic acid (0–90%). The equipment was set to operate at a voltage of 3.5 kV at the ionization source. The data-acquisition range was set to 100 to 2000 *m*/*z* and was processed using the MassLynx v.4.1 software (Waters Corporation, Milford, MA, USA). The deconvolution of the multicharged spectra was performed with the MaxEnt3 with the maximum entropy protocol [74]. Processed spectra were manually sequenced, and the obtained sequence was assembled by overlapping peptides obtained from different digests.

#### 4.2.2. Gene Sequencing

Specific primers were designed starting from the reverse translated partial lectin sequence using the Primer3-web tool [75]. The primers were as follows: forward—3′-TGATCCTTCAAGGTGACGCC-5′; reverse—3′-AGAGAATGGTATTGGTTTCTTTGT-5′. Phusion High-Fidelity DNA Polymerase (ThermoFisher Scientific, Waltham, MA, USA) was used for PCR amplification, and the reaction was set up according to the supplier guidelines for a final reaction volume of 20 µL. The template DNA was extracted from *C. villosa* seeds using the CTAB protocol [76]. The following program was adopted: initial denaturation (95 °C—2 min), denaturation (95 °C—30 s), annealing (60 °C—30 s), amplification (72 °C—30 s), and final amplification (72 °C—5 min), with 35 cycles of amplification.

The amplicons were inserted into a pJET blunt end cloning vector (ThermoFisher Scientific, Waltham, MA, USA) following the manufacturer’s protocol. *Escherichia coli* Top10 cells were transformed via heat shock transformation and grown on carbenicillin-containing LB broth for the selection of transformed cells. Transformation of clones was further confirmed via colony PCR. Recombinant plasmids were purified with the QIAprep Spin Miniprep Kit (QIAGEN, Hilden, Germany). Plasmids were sent for sequencing at LGC Genomics Technology facility, using 3730XL DNA Analyzer System equipment (ThermoFisher Scientific, Waltham, MA, USA). The sequencing files from both forward and reverse reads were analyzed via the Phred v. 0.020425/Phrap v. 1.090518/Consed v. 29.0 softwares [77]. The resulting contigs were treated with the VecScreen tool (NCBI) and translated using the Translate Tool from the ExPASy platform. The combination of gene-sequencing and mass spectrometry data allowed the assembly of the complete lectin sequence which was compared to other protein sequences in the non-redundant databases using the BLASTp alignment tool (NCBI). Cvill contig sequences were deposited in the GenBank database under the accession numbers MW677574 and MW677575 and the protein sequence data reported in this paper will appear in the UniProt Knowledgebase under the accession number C0HM82.

### 4.3. Crystal Structure

#### 4.3.1. Lectin Crystallization

Cvill at 12.5 mg/mL concentration in 0.025 M Tris-HCl buffer pH 7.6 (unliganded and in the presence of 5 mM α-methyl-D-mannopyranoside–α-mm) was applied in screening trials for crystallization. The following crystallization kits were applied: Crystal screen I and II (Hampton Research, Aliso Viejo, CA, USA) and the JBScreen JCSG++ (Jena Bioscience, Jena, Germany). Experiments were set with the hanging drop technique in 96-well crystallization plates. Initial screening was assembled using the Mosquito^®^ system (TTP Labtech, Royston, UK). The plates were sealed with ViewDrop™ II film (TTP Labtech, Royston, UK) and kept at room temperature. The best conditions were optimized for pH and concentration of precipitating agents.

#### 4.3.2. Data Acquisition

Crystal diffraction took place at the Brazilian Synchrotron Light Laboratory (LNLS, Campinas, Brazil). Crystals were captured from the solution with cryoloops (Hampton Research) and dipped in 30% glycerol to avoid ice crystal formation. Afterwards, crystals were taken to the MX2 beamline, where the diffraction with hard X-rays (5 to 15 keV) occurred at 100 K. The crystal exposure time and resolution were adjusted manually. The images were collected using the PILATUS 2M detector (DECTRIS, Baden, Switzerland). A total of 360 images for each crystal were obtained. Each image was collected by oscillating the crystal at an angle of 0.5 degrees, thus covering 180° of crystal rotation.

#### 4.3.3. Data Processing

X-ray diffraction data were analyzed using the CCP4 software package v. 8.0 [78]. Image spots were integrated and indexed via the iMOSFILM software v. 7.4.0, and the best spatial group was defined via POINTLESS v. 1.11.17. The lectin structure was resolved via molecular replacement, using the MOLREP software v. 11.0 [79]. The atomic coordinates of ConGr complexed with X-Man (PDB: 4L8Q) were used as a molecular replacement model. Refinement was carried out in PHENIX [80], Coot was used for model building [81], and the final model was validated in the PDB Validation Tool [82]. The 3D model of Cvill in complex with α-mm was deposited in PDB under the identification number 8SZO. The number of polar and hydrophobic interactions of Cvill with α-mm was evaluated via the software CONTACT v. 1.12.88. Figures were built using PyMOL (Schrödinger LLC, New York, NY, USA) and LIGPLOT v. 2.2 [83]. The binding site volume was calculated using the PyMOL plugin PyVOL [84], with the binding pocket position determined based on the ligand coordinates.

#### 4.3.4. Glycan Microarrays

Glycan array analyses were performed using a synthetic library of glycan of different origins including mammalian, invertebrates, and plants, prepared via chemoenzymatic synthesis as previously described [85]. For the microarray incubation, Cvill was labeled with Alexa FluorTM 555 succinimidyl ester (NHS) (Fisher Scientific) following the manufacturer’s instructions. The labeled lectin was prepared in 25 mM Tris-HCl pH 7.5 solution containing 150 mM NaCl, 5 mM CaCl_2_, 5 mM MgCl_2_, and 0.01% Tween-20 (*v*/*v*) at a final concentration of 0.3 mg/mL. Cvill was incubated in the array for 1 h at room temperature in the dark. After washing the slide with binding buffer and water, the fluorescence was measured using an Agilent G2565BA scanner (Agilent, Santa Clara, CA, USA). The ProScanArray Express software v. 4.0 (Perkin Elmer, Waltham, MA, USA) was used for image processing and the results were represented in Relative Fluorescence Units (RFU), averaging four replicate spots with the standard deviation. The glycan microarray library is illustrated in Supplementary Figure S5.

### 4.4. Molecular Docking

Dockings were performed using the lowest energy conformation of complex and high-mannose N-glycans downloaded from the N-glycan library of the GLYCAM-Web portal (https://glycam.org/ accessed on 24 March 2023). All glycans selected for this work are known components of HeLa cells glycocalyx [30] and/or structures from the glycan array. The binding site was defined as the region within an 11 Å radius from the coordinates of α-mm positioned at the carbohydrate-recognition domain (CRD) in the experimental structure. The simulations were performed using GOLD v. 5.5 (CDCC, Cambridge, UK). For the runs, the protein was considered rigid, and the ligands were made flexible. Interaction scores were calculated via the CHEMPLP scoring function [86,87,88] in which a more negative score indicates a more favorable binding. Best ligand conformations were selected considering the scoring results and geometric parameters. Figures were created with PyMOL.

### 4.5. Antiproliferative Assays

#### 4.5.1. Cell Culture

The antiproliferative activity of Cvill against HeLa, HT1080, and NHDF cells, all obtained from the American Type Culture Collection (Manassas, VA, USA), was studied in vitro. All cell lines were cultured in DMEM High-glucose supplemented with 10% Fetal Bovine Serum (FBS) and 1% penicillin/streptomycin. Cell incubation was always carried out at 37 °C, with an atmosphere of 5% CO_2_ in a humidified incubator.

#### 4.5.2. Cell Viability Assays

Cells were seeded at a density of 1 × 10^4^ cells per well in 96-well plates and treated with various concentrations of Cvill in sterile PBS buffer (pH 7.4) ranging from 0 to 250 μg/mL. Cell viability was checked at 24, 48, and 72 h for HeLa and HT1080 cells and 24 h and 48 h for NHDF cells. Viability was accessed by adding 10 µL of Presto Blue™ Viability Reagent (Invitrogen, Carlsbad, CA, USA) to each well and incubating in the dark for 30 min. Fluorescence was measured at 560/590 nm using a Tecan plate reader (Tecan Group Ltd, Männedorf, Switzerland). Cell viability was expressed in function of the protein concentration and incubation time. For these experiments, 3 technical replicates with 3 biological replicates were applied for HeLa and 4 technical replicates with 2 biological replicates for HT1080 and NHDF. To investigate whether the carbohydrate-binding activity of Cvill is important for its cytotoxic effect, viability assays were also performed with medium containing α-methyl-D-mannopyranoside at a concentration of 100 mM. Cell images were taken using a widefield microscope at 20× magnification.

#### 4.5.3. Reactive Oxygen Species (ROS) Production

ROS generation was measured using 2,7-dichlorofluorescein diacetate (H2DCFDA) (Sigma-Aldrich, St. Louis, MO, USA). HeLa cells were seeded at 1 × 10^4^ cells per well in a 96-well plate. Three different doses of Cvill were tested (125 μg/mL, 31.25 μg/mL, and 7.8 μg/mL). After 48 h incubation, the medium was carefully removed and 100 μL of the H2DCFDA working solution, prepared at 5 μM in sterile PBS, was added to the wells. Plates were then incubated for 30 min in the dark. Wells containing cells without the lectin were used as negative controls, whereas positive controls contained cells treated with 100 μM of hydrogen peroxide for 15 min prior to ROS measurement. Fluorescence measurements were performed in a Tecan microplate reader using wavelengths of 485 nm for excitation and 538 nm for emission [89].

#### 4.5.4. Analysis of Caspase Activation

Caspase 3/7, 8, and 9 activations were assessed using Cell Meter™ Multiplexing Caspase 3/7, 8 and 9 Activity Assay Kit (AAT Bioquest, Sunnyvale, CA, USA). Three different concentrations of the lectin (125 μg/mL, 31.25 μg/mL, and 7.8 μg/mL) were applied. After 48 h incubation, 100 μL of caspase working solution was added to each well followed by a new 30 min incubation. Fluorescence was read at excitation/emission wavelengths of 535/620 nm for Caspase-3/7, 490/525 nm for Caspase 8, and 360/470 nm for caspase 9, in accordance with the manufacturer’s protocol. Fluorescence was normalized via cell viability using PrestoBlue^TM^ Cell Viability Reagent.

### 4.6. Expression Level of Apoptosis and Autophagy-Related Genes

For qPCR experiments, HeLa cells (5 × 10^4^ cells per well) were cultivated in 24-well plates and incubated with different concentrations of Cvill (125 μg/mL, 31.25 μg/mL and 7.8 μg/mL) for 48 h. The control experiment contained cells incubated in the absence of the lectin. Experiments were executed with three biological replicates. RNA was isolated using the TRI-Reagent (Sigma-Aldrich) optimized for cells cultured in monolayers and all samples were treated with DNAse I (Sigma-Aldrich). cDNA was synthesized starting from 1000 ng of RNA using M-MLV Reverse transcriptase enzyme (ThermoFisher Scientific). RT-qPCR was performed with three technical replicates for each biological replicate. Reactions were prepared with 0.5 μM of each primer, 10 μL of SYBR^®^ Green SuperMix (Bio-Rad, Hercules, CA, USA), and 1 μL of cDNA in a total volume of 20 μL per well. The PCR program included one initial step of 95 °C for 10 min and 42 cycles of 95 °C for 15 s, 60 °C for 25 s, and 72 °C for 20 s. Experiments were executed in a Bio-Rad CFX96 qPCR thermocycler instrument using the Bio-Rad CFX Manager 3.1 software. The transcript level for genes related to apoptotic mechanisms (BAX, BAK, FADD and PUMA) and autophagy pathways (LC3) was evaluated using Relative Expression Software Tool (REST 2009) v. 2.0.13 [90]. GAPDH and HPRT1 were used as reference genes. The Bootstrap method was used to analyze the significance of the gene expression using 95% confidence intervals and 2000 randomizations. Primers are listed in the Supplementary Figure S8.

### 4.7. Subcellular Localization of Cvill

Confocal microscopy was employed to check the subcellular location of Cvill in HeLa cells. For this, the lectin was fluorescently tagged using Tetramethylrhodamine (TRITC, Sigma-Aldrich) in 100 mM sodium bicarbonate buffer pH 9.0. HeLa cells were cultivated overnight in glass bottom dishes at 35,000 cells per dish. Afterwards, cell culture medium was discarded, cells were washed with PBS, and new media containing Cvill-TRITC at 125 μg/mL and 31.25 μg/mL were added to dishes, followed by a 48 h incubation period. Cells were stained with Calcein (Thermo Fisher Scientific, Waltham, MA, USA) for 45 min in a cell incubator and washed twice with PBS before confocal microscopy experiments on a Nikon Instrument A1 Confocal Laser Microscope. Experiments were performed in triplicates and about 140 cells were analyzed individually. NIS-Elements software v. 4.60 (Nikon, Tokyo, Japan) was used for data collection and image analysis. The software NIS-Elements Viewer v. 5.21 and ImageJ v. 2.14.0 (NIH, Bethesda, MD, USA) were used to make the figures.

### 4.8. Statistical Analysis

Statistical analyses of cytotoxicity, ROS, and caspase assays were performed using the GraphPad Prism 6.01 software. Statistical significance was determined by analysis of variance (ANOVA) and Tukey’s multiple comparison test. IC50 was calculated via non-linear regression. Significance was indicated by *p*-values, with (*p* < 0.01) unless another *p*-value value in the text is explicitly indicated. The statistical analysis of the qPCR data was performed via the REST 2009 software v. 2.0.13. Significance was given via the Bootstrap method using 95% confidence intervals.

## Figures and Tables

**Table 1:** Data collection, refinement, and structure quality parameters.

PDB ID 8SZO	
Parameters	Values
Data collection	
Space group	I222
Unit cell parameters	61.49 Å; 84.44 Å; 89.07 Å
	90.00°; 90.00°; 90.00°
Number of reflections	40,674 (5399) ^d^
Number of unique reflections	8301 (1200) ^d^
Molecules per asymmetric unit	1
Resolution limits	27.48–2.50 (2.64–2.50) ^d^
*R_merge_* ^a^ (%)	11.2 (43.3) ^d^
Completeness (%)	99.6 (99.7) ^d^
Multiplicity	4.9 (4.5) ^d^
Average I/6(I)	8.9 (3.0) ^d^
Wilson B-factor (Å^2^)	43.9
Molecular replacement	
wRfactor	0.431
Score	0.6826
Refinement	
Resolution range	27.48–2.50
*R_factor_* ^b^ (%)	19.80 (25.28) ^d^
*R_free_* ^c^ (%)	26.70 (35.84) ^d^
Number of reflections	8295 (808) ^d^
Reflections used in Rfree	408 (34) ^d^
Number of residues in asymmetric unit	231
Number of water molecules	88
Variations of RMS Optimal Values	
Bond length (Å)	0.009
Bond angles (degree)	1.055
Temperature factors	
Average B value for whole protein chain	46.0
Ligand	50.3
Solvent	49.9
Rotamers and Ramachandran Plot	
Rotamer outliers (%)	0.50
Residues in most favored regions (%)	96.51
Residues in additional allowed regions (%)	3.49
Residues in generously allowed regions (%)	0

^a^ $Rmerge=\frac{\Sigma_{hkl}\Sigma_{j}\left|I_{hkl,j}-\langle I_{hkl}\rangle \right|}{\Sigma_{hkl}\Sigma_{j}I_{hkl,j}}$, ^b^ $Rfactor=\frac{\sum \left|F\;obsv-F\;calc\right|}{\sum \left|F\;obsv\right|}$, ^c^ Rfree was calculated using a random subset of 5% of reflections excluded from refinement, ^d^ values in parentheses represent the OuterShell.

**Table 2:** Interactions between Cvill and α-methyl-mannopyranoside.

Cvill Residues and Atoms	Ligand Atom	Distance (Å)
Polar interactions		
Asn14/ND2	α-mm/O4	2.50
Gly98/N	α-mm/O6	3.39
Leu99/N	α-mm/O5	3.28
Asp208/OD1	α-mm/O6	3.39
Asp208/OD1	α-mm/O4	2.85
Asp208/OD2	α-mm/O6	2.39
Met228/N	α-mm/O3	2.78
Van der Waals interactions		
Gly98/CA	α-mm/O6	3.25
Ala207/CB	α-mm/O6	3.21
Asp208/CG	α-mm/O6	3.20
Asp208/OD2	α-mm/C4	3.40
Gly227/CA	α-mm/O3	3.33
Met228/CG	α-mm/O3	3.37
Met228/CE	α-mm/C3	3.10
Met228/CE	α-mm/O3	2.75

Source: Interactions calculated via CONTACT software v. 1.12.88 (CCP4 v. 8.0).

## Data Availability

Data are available upon reasonable request.

## Supplementary Materials

The following supporting information can be downloaded at: https://www.mdpi.com/article/10.3390/ijms242115966/s1.
