# Supplementary material for: Structural Analysis and Characterization of an Antiproliferative Lectin from Canavalia villosa Seeds
Source: Int J Mol Sci. 2023 Nov 4;24(21):15966. doi: 10.3390/ijms242115966 (PMC10649158; doi:10.3390/ijms242115966)
Supplement: Supplementary file 1 [file ijms-24-15966-s001.zip › ijms-2638525-supplementary.pdf]

```
Contig1     ttgatccttcaaggtgacgccacaactgattcagatggtaacttacaactcacaagggtg    60
Contig2     ttgttccttcaaggtgacgccacagctgattcagatggtaacttacaactcacaagggtg    60
proCvill     L  I  L  Q  G  D  A  T  T  D  S  D  G  N  L  Q  L  T  R  V     20

Contig1     tcaagtaatgggactccacagggaaacagtatgggccgggctttgttctacgccccagtc    120
Contig2     tcaagtaatgggactccacagggaaacagtgtgggccgggctttgttctacgccccagtc    120
proCvill     S  S  N  G  T  P  Q  G  N  S  V  G  R  A  L  F  Y  A  P  V     40

Contig1     catatttgggaaagttctgctgtggtggcaagctttgacgccacctttacatttctcata    180
Contig2     catatttgggaaagttctgctgtggtggcaagctttgacgccacctttacatttctcata    180
proCvill     H  I  W  E  S  S  A  V  V  A  S  F  D  A  T  F  T  F  L  I     60

Contig1     aagtcacccgacagggatccagctgatggaattaccttcttcatttcaaatatggacagt    240
Contig2     aagtcacccgacagggatccagctgatggaattaccttcttcatttcaaatatggacagt    240
proCvill     K  S  P  D  S  D  P  A  D  G  I  T  F  F  I  S  N  M  D  S     80

Contig1     accctccctatgggttccactggaatgcttcttggactcttccctgatgctaatgttatc    300
Contig2     accctccctatgggttccactggaatgcttcttggactcttccctgatgctaatgttatc    300
ProCvill     T  L  P  R  G  S  T  G  M  L  L  G  L  F  P  D  A  N  V  I     100

Contig1     aaaaattccaccaatcttgatttcaacgctgcttataat------tccgatactattgtt    354
Contig2     aaaaattccaccaatcttgatttcaacgctgcttataattccaattccgatactgttgtt    360
proCvill     K  N  S  T  N  L  D  F  N  A  A  Y  N  S  N  S  D  T  I  V     120

Contig1     gctgttgaattggatacctatcccaatactgatattggagatccaaattatccacacatt    414
Contig2     gctgttgaattggatacctatcccaatcctgatattggagatccaaattatccacacatt    420
ProCvill     A  V  E  L  D  T  Y  P  N  T  D  I  G  D  P  N  Y  P  H  I     140

Contig1     ggtatcgatatcaaatctattcgctccaagaaaaccgctaggtggaacatgcaaaatgga    474
Contig2     ggtatcgatatcaaatctattcgctccaagaaaaccgctaggtggaacatgcaaaatgga    480
proCvill     G  I  D  I  K  S  I  R  S  K  K  T  A  R  W  N  M  Q  N  G     160

Contig1     aaggtaggcacggcacatatcacctataactctgtttgtaagagactaagtgccattgtt    534
Contig2     aaggtaggcactgcacacatcatctataactctgttggtaagagactaagtgccattgtt    540
proCvill     K  V  G  T  A  H  I  I  Y  N  S  V  G  K  R  L  S  A  I  V     180

Contig1     tcttatcctaacggtgactctgccaccgtctcttatgacgttgacctcgacaatgtcctt    594
Contig2     tcttatcctaacggtgactcttccaccgtctcttatgacgttgacctcgacaatgtcctt    600
proCvill     S  Y  P  N  G  D  S  A  T  V  S  Y  D  V  D  L  D  N  V  L     200

Contig1     cctgaatgggttagagttgggctttctgcttcaaccggactcaacaaagaaaccaatacc    654
Contig2     cctgaatgggttagagttgggctttctgcttcaaccggactcaacaaagaaaccaatacc    660
proCvill     P  E  W  V  R  V  G  L  S  A  S  T  G  L  Y  K  E  T  N  T     220

Contig1     attctc                                                          660
Contig2     attctc                                                          666
proCvill     I  L                                                           222
```

Supplementary Figure S1. Nucleotide sequence of two contigs of Cvill gene and the translated amino acid sequence of the lectin precursor. If more than one amino acid could becoded in the same position due to isoform variations, the most commonly found residue in MS/MSdata was used to depict the translated sequence. The region highlighted in gray is lost during posttranslational processing and the remaining regions are thereafter reconnected in reverse order to assemble the mature Cvill.

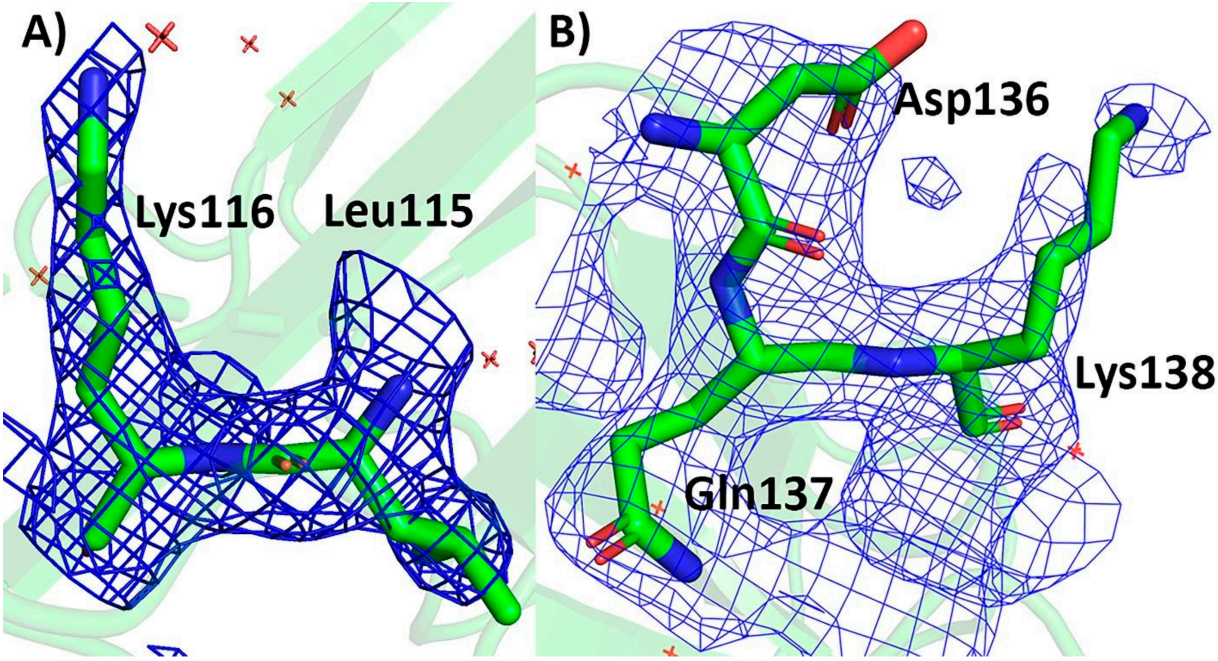

Supplementary Figure S2. Fit of residues Leu115-Lys116 (A) and Asp136-Gln137-Lys138 (B) in the electron density map of Cvill. The electron density map, along with mass spectrometry digestion pattern, and sequence identity with other Diocleinae lectins were used to solve the sequencing information of these two small fragments.

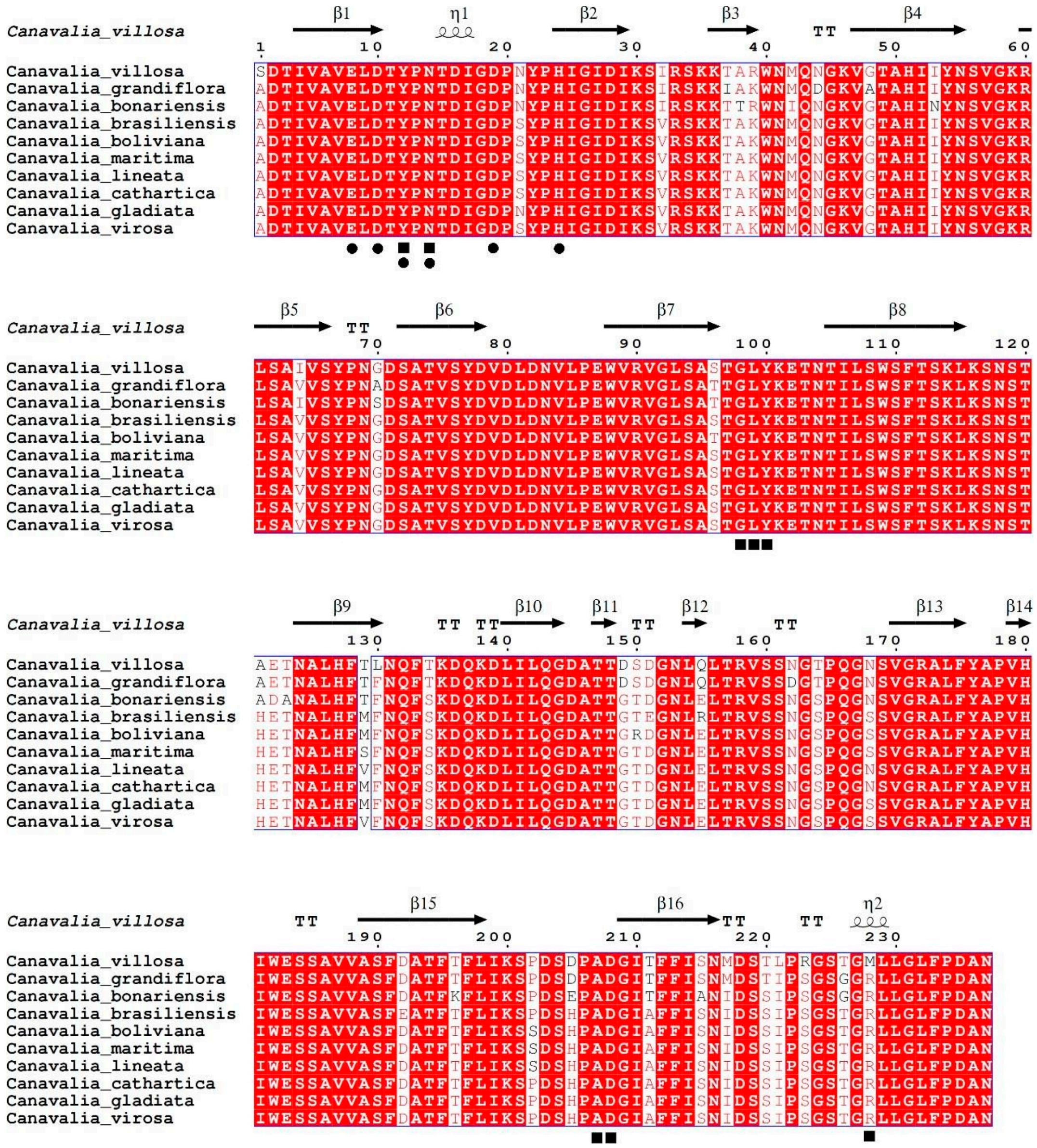

Supplementary Figure S3. Alignment of Cvill with *Canavalia* lectins. Residues involved in the Carbohydrate Recognition Domain (CRD) are represented by a square, while residues involved in the Metal Binding Site are marked with a circle. Sequence alignment was performedusing the online tool ClustalW. Sequence similarities and secondary structure prediction were obtained using ESPript 3.0.

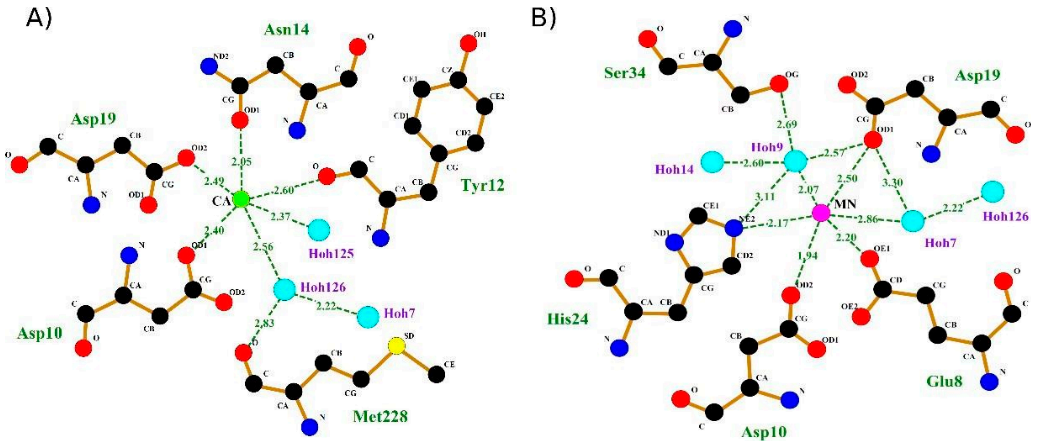

Supplementary Figure S4. Graphical representation of the octahedral coordination of calcium (A) and manganese (B) ions in the metal binding site of Cvill.

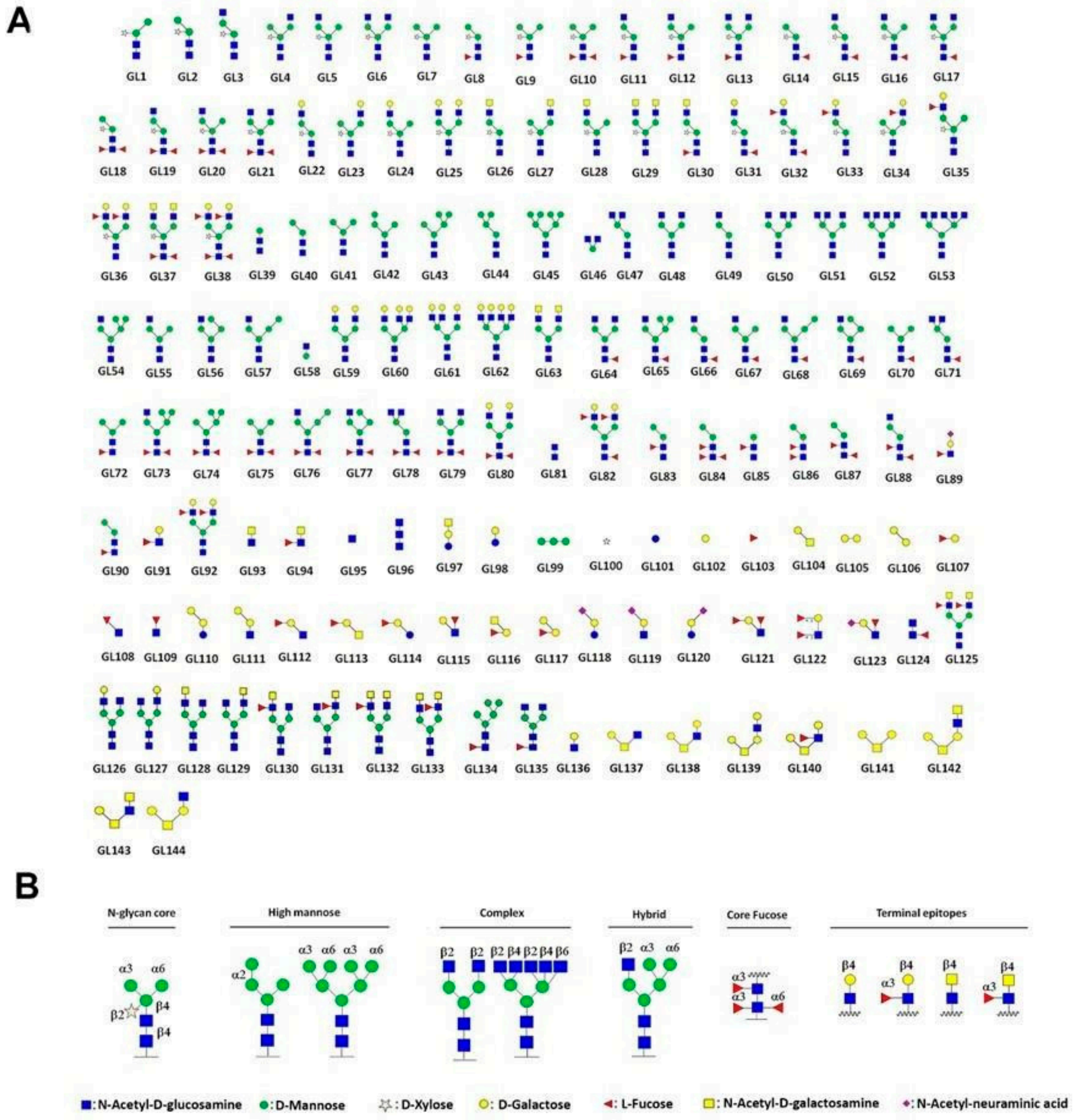

Supplementary Figure S5. Glycan library used in the array experiments. (**A**) Glycan structures included on the microarrays, (**B**) Nature of the glycosidic linkages of the N-glycan structures on the microarrays.

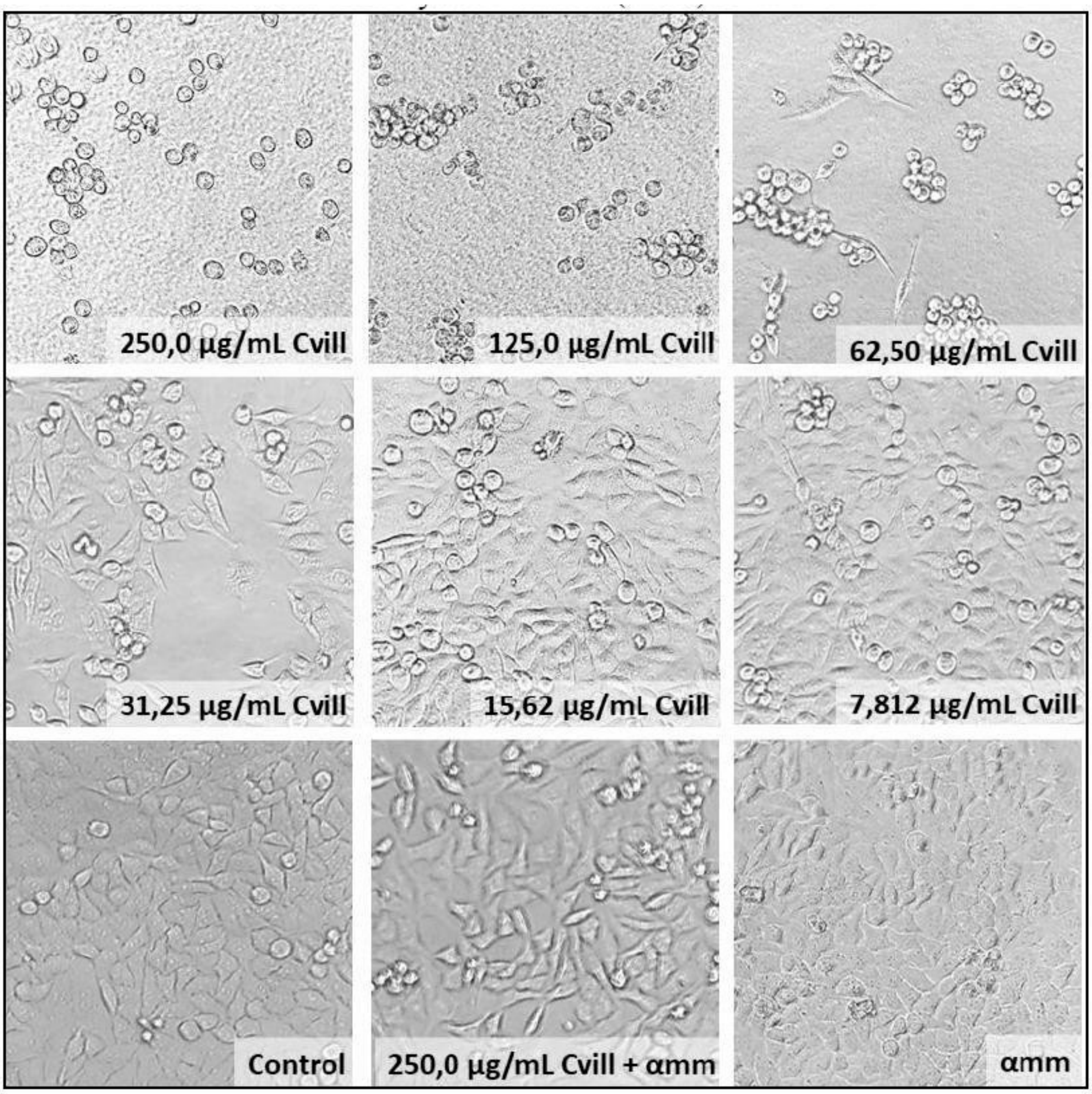

Supplementary Figure S6. Morphology of HeLa cells in presence and absence of lectin and α-methyl-D-mannopyranoside. Images were taken at 20x magnification.

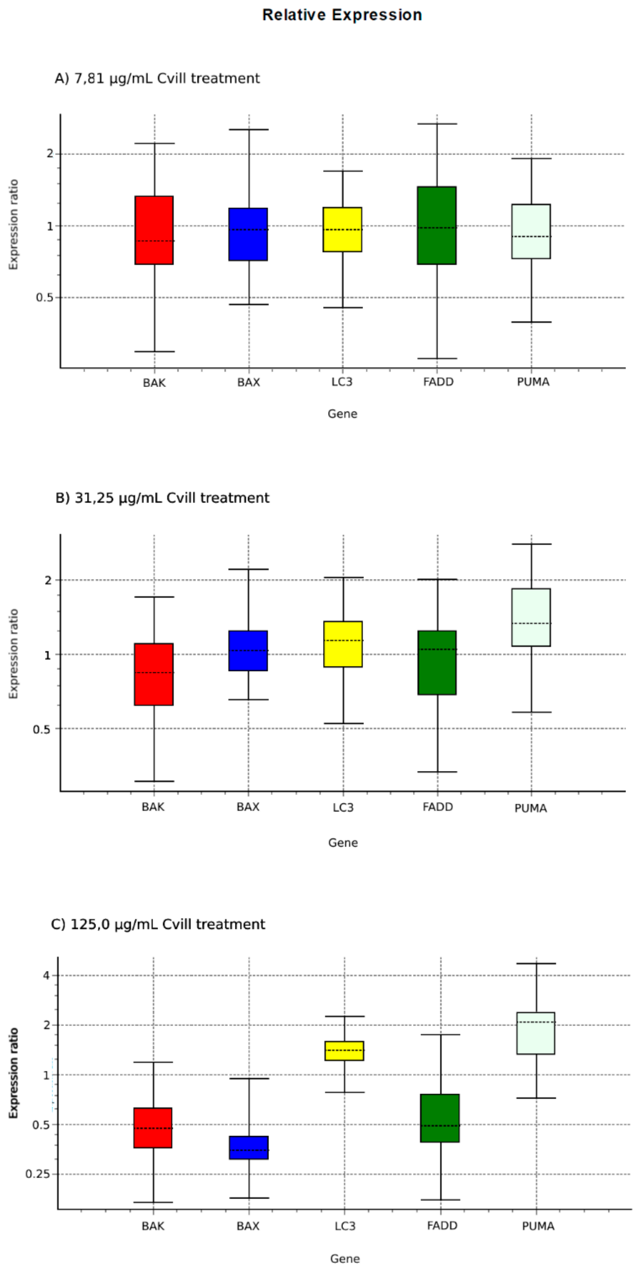

Supplementary Figure S7. Relative expression level of genes related to cell death in HeLa cells treated with Cvill at 7.81 µg/mL (A), 31.25 µg/mL (B), and 125.0 µg/mL (C), concentrations for the incubation period of 48 h. The box diagram provides the visualization of data asymmetry and dispersion, in which 50% are distributed within the boxes and 50% are within the range delimited by the lines. The dashed line within the boxes represents the median of the expression ratio values. Boxes that move upwards from the ratio value equal to 1 indicate increased expression in relation to the control, while those which move downwards indicate decreased expression. The two reference genes HPRT1 and GADPH were used to normalize the expression data. The statistics were calculated based on a 95% confidence interval considering 2000 randomizations. The graphics were generated using the REST 2009 program.

Gene	Primers
Apoptosis	
PUMA	Fw: 5' ATGCCTGCCTCACCTTCATC 3'<br>Rv: 5' TCAGCCAAAATCTCCCACCC 3'
BAX	Fw: 5' AGTAACATGGAGCTGCAGAGG 3'<br>Rv: 5' ATGGTTCTGATCAGTTCCGG 3'
BAK	Fw: 5' ACTCTACCCCTGCTCCCATT 3'<br>Rv: 5' CTTGGAGGCTTCTGACACGT 3'
FADD	Fw: 5' GTGCGGGAGTCACTGAGAAT 3'<br>Rv: 5' CTCCTGTTCTGGAGGTCACG 3'
Autophagy	
LC3	Fw: 5' AGAAGGCGCTTACAGCTCAA 3'<br>Rv: 5' AGATTGGTGTGGAGACGCTG 3'
Reference genes	
GAPDH	Fw: 5' GAAGGTGAAGGTCGGAGTC 3'<br>Rv: 5' GAAGATGGTGATGGGATTTC 3'
HPRT1	Fw: 5' TGAGGATTTGGAAAGGGTGTT 3'

Supplementary Figure S8. Primers applied in the qPCR experiments.
